# Aβ43 in human Alzheimer’s disease: effects of active Aβ42 immunization

**DOI:** 10.1186/s40478-019-0791-6

**Published:** 2019-09-02

**Authors:** Lieke Jäkel, Delphine Boche, James A. R. Nicoll, Marcel M. Verbeek

**Affiliations:** 1 Department of Neurology, Donders Institute for Brain, Cognition and Behaviour, Radboud Alzheimer Center, Radboud University Medical Center, Nijmegen, The Netherlands; 2 Department of Laboratory Medicine, Radboud University Medical Center, Nijmegen, The Netherlands; 3 Clinical Neurosciences, Clinical and Experimental Sciences, University of Southampton, Southampton, UK; 4 Department of Cellular Pathology, University Hospital Southampton NHS Foundation Trust, Southampton, UK

**Keywords:** Amyloid-β, Aβ43, Cerebral amyloid angiopathy, Alzheimer’s disease, Aβ immunotherapy, Human study, Immunohistochemistry

## Abstract

Neuropathological follow-up of patients with Alzheimer’s disease (AD) who participated in the first clinical trial of Amyloid-β 42 (Aβ42) immunization (AN1792, Elan Pharmaceuticals) has shown that immunization can induce removal of Aβ42 and Aβ40 from plaques, whereas analysis of the cerebral vessels has shown increased levels of these Aβ peptides in cerebral amyloid angiopathy (CAA). Aβ43 has been less frequently studied in AD, but its aggregation propensity and neurotoxic properties suggest it may have an important pathogenic role. In the current study we show by using immunohistochemistry that in unimmunized AD patients Aβ43 is a frequent constituent of plaques (6.0% immunostained area), similar to Aβ42 (3.9% immunostained area). Aβ43 immunostained area was significantly higher than that of Aβ40 (2.3%, *p* = 0.006). In addition, we show that Aβ43 is only a minor component of CAA in both parenchymal vessels (1.5 Aβ43-positive vessels per cm^2^ cortex vs. 5.3 Aβ42-positive vessels, *p* = 0.03, and 6.2 Aβ40-positive vessels, *p* = 0.045) and leptomeningeal vessels (5.6% Aβ43-positive vessels vs. 17.3% Aβ42-positive vessels, *p* = 0.007, and 27.4% Aβ40-positive vessels, *p* = 0.003). Furthermore, we have shown that Aβ43 is cleared from plaques after Aβ immunotherapy, similar to Aβ42 and Aβ40. Cerebrovascular Aβ43 levels did not change after immunotherapy.

## Introduction

Alzheimer’s disease (AD) is characterized by the extracellular accumulation of the Amyloid-β (Aβ) protein in the brain parenchyma as plaques and in cerebrovascular blood vessel walls as cerebral amyloid angiopathy (CAA), and the intraneuronal accumulation of the tau protein. It is hypothesized that CAA is caused by dysfunctional Aβ elimination: a process that may include enzymatic degradation, receptor-mediated clearance across the blood-brain barrier (BBB), and drainage with interstitial fluid along perivascular pathways [33]. Blockage of these pathways may lead to aggregation of Aβ in the vessel walls as CAA [10, 22]. In a subset of CAA patients, Aβ deposits in both arteries and arterioles, and capillaries (CAA type 1), whereas more commonly, cerebrovascular Aβ accumulation is restricted to larger vessels, without affecting capillaries (CAA type 2) [34]. The occipital lobe is most frequently and severely affected by CAA [1, 2], and leptomeningeal vessels are more frequently affected compared to parenchymal vessels [1, 27].

Aβ is a peptide consisting of 38 to 43 amino acids, with Aβ40 and Aβ42 being the most-studied Aβ isoforms. Aβ42 is a major constituent of plaques, whereas some studies show that Aβ40 is the predominant species in CAA [9, 28]. Aβ is produced through cleavage of the amyloid precursor protein (APP) by γ-secretase, which is predominantly initiated at ε cleavage sites situated after Leu49 or Thr48. The resulting Aβ49 or Aβ48 are sequentially cleaved in increments of three amino acids, which leads to the production pathways Aβ49 → Aβ46 → Aβ43 → Aβ40 and Aβ48 → Aβ45 → Aβ42 [7, 26, 32]. Only relatively recently it was found that in addition to Aβ40 and Aβ42, also Aβ43 may play a major role in AD, as plaques contain more Aβ43 than Aβ40 [13, 29, 36]. Interestingly, although Aβ43 seems to be a major component of plaques, no significant levels of Aβ43 have been reported in CAA [13, 36].

The amyloid cascade hypothesis states that aggregation of Aβ into plaques is the initial event in AD, followed by the formation of neurofibrillary tangles, and synaptic and neuronal loss leading to cognitive decline [17]. Therefore, in the past two decades, much attention has been directed towards the possibilities of Aβ immunotherapy to remove Aβ from the brain. Both active and passive Aβ immunotherapy approaches have been shown to be effective in the removal of plaques from the human brain, although so far the effects on slowing or preventing cognitive decline have been mainly disappointing [12, 31, 35].

In the first clinical trial of Aβ immunization, which started in 2000, patients with mild to moderate AD were actively immunized with synthetic full-length Aβ42 (AN1792, Elan Pharmaceuticals [4]). *Post-mortem* neuropathological examination of 12 immunized AD patients showed variable, sometimes extensive, clearance of Aβ plaques from the cerebral cortex [5, 20, 23, 30]. Interestingly, decreases in parenchymal amyloid were associated with an increased vascular amyloid burden [21, 23, 24]. This lead to the hypothesis that immunization results in solubilization of Aβ plaques, followed by perivascular drainage of the solubilized Aβ that leads to the development of CAA [5].

In this study, we have investigated the expression of Aβ43 in parenchymal plaques and CAA in the AD brain. Furthermore, we assessed the effects of Aβ42 immunotherapy on Aβ43 accumulation, both in plaques and the cerebral blood vessels, by *post-mortem* examination of a unique cohort of patients who were included in the first AN1792 clinical trial. Aβ43 deposition was analysed in relation to Aβ40 and Aβ42, to generate more insight into the relative deposition of Aβ43 in plaques and CAA and to study the effects of immunotherapy on the distribution of Aβ43 in AD brains. Analysing the fate of different Aβ isoforms following immunotherapy may provide insight into potential differences in clearance efficiency.

## Materials and methods

### Sample cohort

Sixteen Alzheimer’s disease patients immunized against Aβ42 (AN1792, Elan Pharmaceuticals Inc. [4]), denoted iAD, with confirmed *post-mortem* neuropathological AD diagnosis were examined for the current study. Twenty-one non-immunized AD cases (AD) from the South West Dementia Brain Bank (SWDBB Bristol, UK) were included for comparison. The AD and iAD groups were matched as far as possible for age, gender, and *APOE* genotype, but disease duration differed between the groups (Table 1). The study of the iAD cohort was performed under the ethical approval from Southampton and South West Hampshire Local Research Ethics Committees (Reference No: LRC 075/03/w). The use of the SWDBB tissue was covered by the ethical approval from North Somerset and South Bristol Hampshire Local Research Ethics Committees (Reference No: REC 08/H0106/28 + 5).

**Table 1:** Characteristics of the groups

	AD	iAD	*p*
N	21	16	
Sex (% male)	48	56	0.60^a^
Duration Dementia (years, mean ± sd)	8.5 ± 3.3	11.9 ± 4.5	0.02^b^
	n/a: *n* = 4		
*APOE* genotype	2,2 or 2,3: 15%	2,2 or 2,3: 0%	0.45^a^
	3,3: 15%	3,3: 25%	
	3,4: 55%	3,4: 50%	
	4,4: 15%	4,4: 25%	
	n/a: *n* = 1	n/a: *n* = 4	
Age at death (years, mean ± sd)	78.0 ± 7.5	79.2 ± 8.4	0.64^b^
Survival time after immunization (months, mean ± sd)	N.A.	91.9 ± 57.7	

Abbreviations: *AD* Alzheimer’s disease patients, *iAD* immunized Alzheimer’s disease patients, *Aβ* amyloid-β, *N.A* not applicable. n/a: not available.

Analysed by ^a^Pearsons’ chi-square and ^b^t-test.

### Immunohistochemistry

Four micrometer-thick paraffin sections of the middle temporal gyrus were used for immunohistochemistry. After rehydration and antigen retrieval, including neat formic acid pre-treatment and heat-induced epitope retrieval, sections from AD and iAD cases were stained with rabbit-anti-human Aβ43 (IBL, Fujioka, Japan; cat. no. 18583, 0.5 μg/ml). Furthermore, sections were stained with mouse-anti-human Aβ42 (clone 21F21, 1:4000) and mouse-anti-human Aβ40 (clone 2G3, 1:4000), both provided by Elan Pharmaceuticals (South San Francisco, CA, USA) [16]. Binding of biotinylated secondary antibody (goat-anti-rabbit or rabbit-anti-mouse, DAKO, Glostrup, Denmark) was detected with the Vectastain ELITE ABC kit (Vector Laboratories, Peterborough, UK), using 3,3′ diaminobenzidine (DAB) as chromogen and 0.05% hydrogen peroxide as substrate. Sections were mounted in DePex (BDH Laboratory Supplies, Poole, UK).

### Antibody specificity

We tested the specificity of the antibodies by immunoassays. First, 25 nM of synthetic Aβ43 (Anaspec, Fremont, CA, USA; cat. no. AS-25357), Aβ42 (Bachem, Bubendorf, Switzerland; cat. no. H-1368), and Aβ40 (QCB, Hopkinton, MA, USA; cat. no. 20–1000), all diluted in NaHCO3 (pH 9.6), were coated overnight at 4 °C on a 96-wells plate. Then, the plate was washed 3 times with PBS containing 0.05% Tween20 and blocked 1 h at room temperature (RT) with PBS containing 1% BSA. Wells were then incubated with rabbit-α-Aβ43 (1:500), 21F12 (1:10000), 2G3 (1:3000), or biotinylated pan-Aβ antibody (clone 4G8, Biolegend, San Diego, CA, USA; cat. no. 800701, 1:2500), diluted in PBS containing 1% BSA. After 2 h incubation at RT and subsequent washing, wells were incubated for 1 h with a secondary antibody (goat-α-rabbit HRP, rabbit-α-mouse HRP, or streptavidin-HRP) at RT. As a substrate, 100 μl of 3,3′,5,5′-tetramethylbenzidine (TMB) solution was added, and the reaction was stopped with 50 μl of 1 M H2SO4. Optical density (OD) values were measured at 450 nm using a Tecan Infinity F50 plate reader.

### Quantification of Aβ load

Whole sections were scanned at a 20x objective magnification using a V120 virtual slide microscope (Olympus, Tokyo, Japan). Thirty regions of interest (ROIs) were selected in a zigzag sequence along the cortical ribbon, to ensure representation of cortical layers, in similar anatomical regions for each case. ROIs were analysed using Fiji software (version 1.51) to obtain a protein load defined as percentage of immunostained area, as in previous studies [30, 37]. The immunostained area predominantly represents the amount of Aβ accumulated in the brain parenchyma as plaques, and is therefore referred to as such, although CAA might have made a small contribution to this value.

### Quantification of CAA

Quantification of CAA was performed on the entire section at a 10x objective magnification. The grade of Aβ staining was scored in each leptomeningeal vessel as follows: fully affected by CAA (full circumference and thickness), partially affected by CAA, or not affected by CAA, and the data were expressed as percentage stained vessels (%). In the cortex, blood vessels fully affected by CAA (full circumference and thickness) or partially affected by CAA were counted, and the data were presented as the number of affected vessels per cortical grey matter area (cm^2^), as previously performed in our group [5]. For analyses, fully and partially stained vessels were combined into one group (e.g. ‘positive vessels’), as no differences between fully and partially stained vessels were detected.

### Statistical analysis

Data analysis was performed using IBM SPSS Statistics 25 (Armonk, NY, USA) and GraphPad Prism 5 (La Jolla, CA, USA). Normality of data was assessed using D’Agostino & Pearson test. The protein loads of the three Aβ isoforms were compared using a Kruskall-Wallis test and post-hoc Mann Whitney U-test with Bonferroni correction for multiple analysis. The numbers of vessels in AD patients affected by Aβ43, Aβ42, and Aβ40 were analysed using paired t-tests. Correlations between Aβ isoform length and Aβ load in plaques and CAA was assessed using Spearman’s test. The protein loads and total numbers of positive vessels for each Aβ isoform were compared between AD and iAD cases using two-sample two-sided t-test or nonparametric Mann–Whitney U-test (depending on the normality of the data). The correlations between total numbers of leptomeningeal and parenchymal vessels affected by the three Aβ isoforms were assessed in the combined cohort of AD and iAD patients using Spearman’s test. The threshold for statistical significance was set at 5%.

## Results

### Antibody specificity

**Fig. 1:**
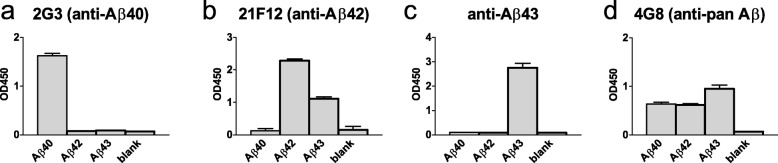
Specificity of anti-Aβ antibodies. The anti-Aβ43 antibody specifically recognizes Aβ43 (**a**). The 21F12 antibody also recognizes Aβ43, in addition to Aβ42 (**b**). The 2G3 antibody is specific for Aβ40 (**c**). Detection with the 4G8 antibody shows that the different Aβ isoforms are present in comparable amounts and bind to the plate with similar affinity (**d**). Data are presented as mean (standard deviation)

The immunoassays showed that the anti-Aβ43 antibody specifically recognized Aβ43 (Fig. 1a). Interestingly, the 21F12 antibody detected both the Aβ42 peptide, and, to a much lesser extent, the Aβ43 peptide (Fig. 1b). The 2G3 antibody was specific for Aβ40 (Fig. 1c). Detection using the pan-Aβ 4G8 antibody was used to confirm that all three Aβ peptides were coated in similar amounts (Fig. 1d).

### Aβ peptides in plaques in AD cases

**Fig. 2:**
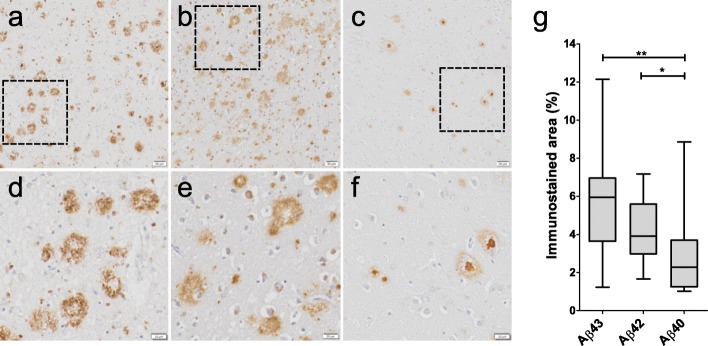
Parenchymal Aβ load in AD cases. Representative images of parenchymal tissue stained by antibodies directed against Aβ43 (**a**), Aβ42 (**b**), and Aβ40 (**c**) at 20x objective magnification. Indicated areas are shown at higher magnification (**d-f**). A significantly larger portion of the cortex is immunostained by the Aβ43- and Aβ42-specific antibodies compared to the Aβ40-specific antibody (**g**). The cases shown in **a-f** are representative of the median immunostained area as plotted in **g**. Box plot shows median values with the 25th and 75th percentile as boundaries and whiskers indicating minimum and maximum values. Scale bar = 50 μm (upper panel) or 20 μm (lower panel). *p ≤ 0.05; **p ≤ 0.01

Qualitative assessment of reactivity of the different Aβ antibodies in the middle temporal lobe showed that anti-Aβ43 and anti-Aβ42 antibodies immunolabeled diffuse and dense-core plaques, whereas Aβ40 detection was largely confined to plaque cores (Fig. 2a-f). Comparison of the area immunostained by each Aβ antibody showed that Aβ43 immunostaining (median 6.0%) covered a larger area of the cortex compared to Aβ40 (median 2.3%, *p* = 0.006). The area covered by Aβ42 immunostaining (median 3.9%) was also significantly higher than the area covered by Aβ40 (*p* = 0.036), but did not differ from the Aβ43 immunostained area (*p* = 0.17; Fig. 2g).

### Aβ peptides in CAA in AD cases

**Fig. 3:**
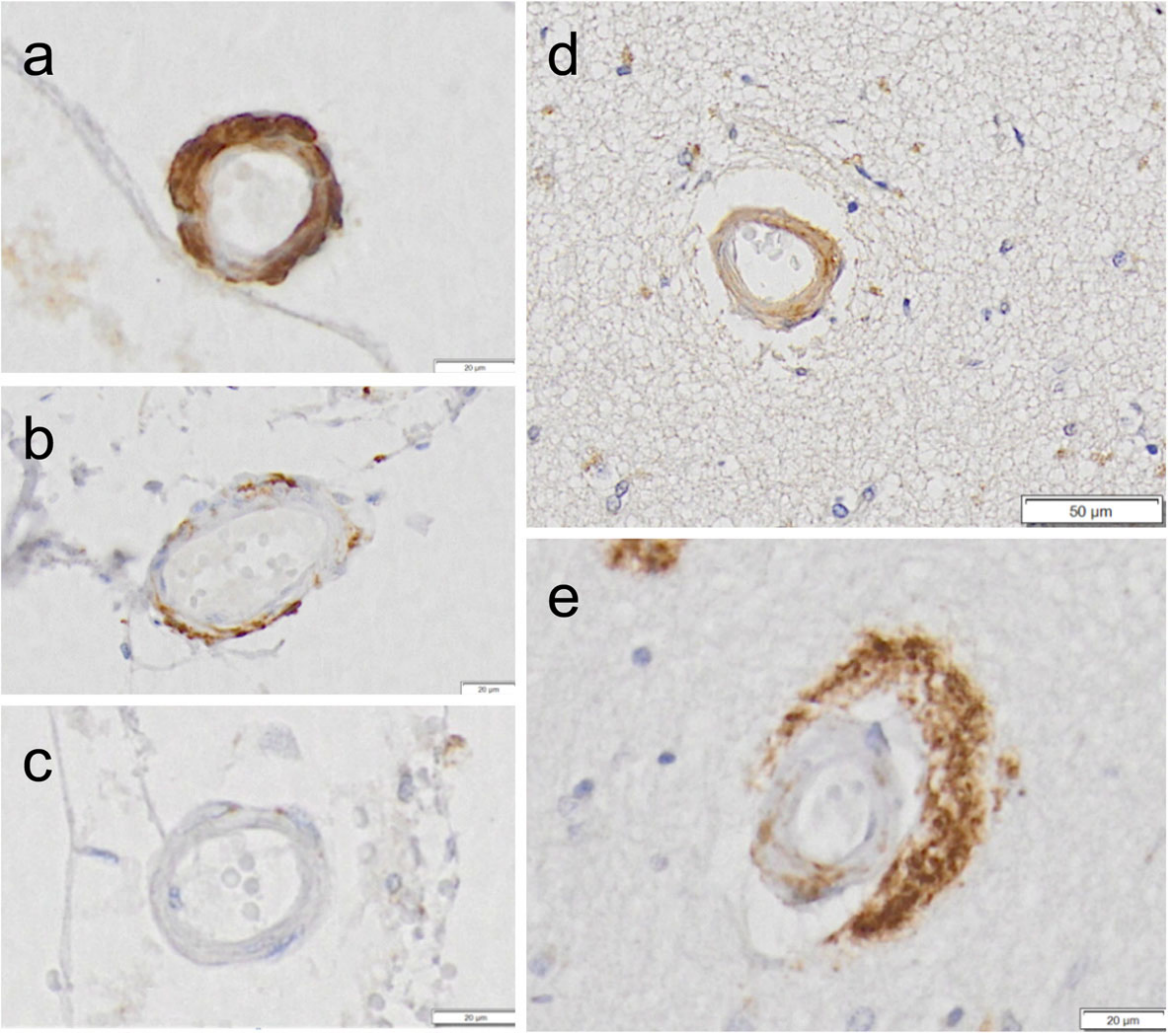
Representative examples of Aβ43 staining in cerebral small vessels. Aβ43-immunostaining was detected in both leptomeningeal (**a, b**) and parenchymal (**d, e**) vessels. Some immunostained vessels were partially stained (**b, e**). In others, Aβ43 staining was detected in the full circumference and thickness of the vessel wall (**a, d**). Many vessels were devoid of Aβ43 (**c**). Scale bar = 20 μm (**a, b, c, e**) or 50 μm (**d**)

**Fig. 4:**
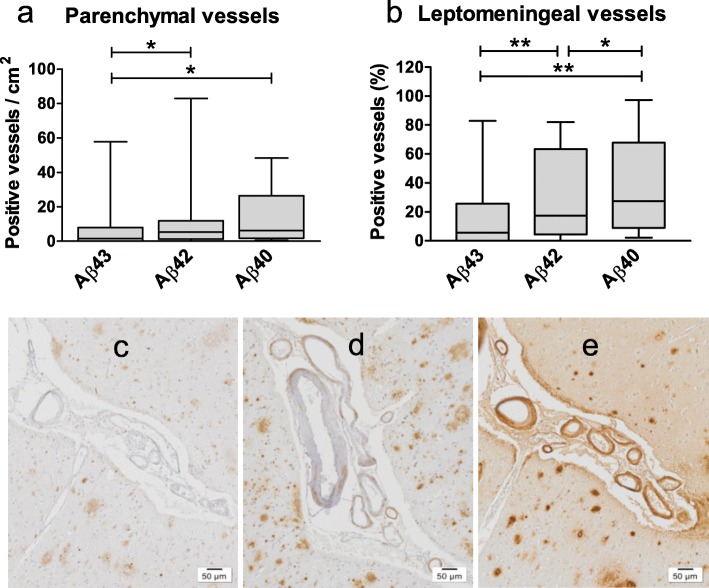
Aβ peptides in the cerebrovasculature of AD cases. Analysis of the numbers of parenchymal (**a**) and leptomeningeal (**b**) vessels affected by Aβ43, Aβ42, and Aβ40 in AD cases shows significantly lower numbers of vessels affected by Aβ43 compared to the other peptides. Images of cerebrovascular immunostaining of Aβ43 (**c**), Aβ42 (**d**), and Aβ40 (**e**) in the same cortical area of an AD case. Box plots show median values with the 25th and 75th percentile as boundaries and whiskers indicating minimum and maximum values. Scale bar = 50 μm. *p ≤ 0.05; **p ≤ 0.01

Aβ43 was, like Aβ42 and Aβ40, detected in leptomeningeal and parenchymal blood vessels as illustrated in Fig. 3. Quantification of vascular staining revealed lower numbers of parenchymal vessels affected per cm^2^ by Aβ43 (median 1.5) compared to Aβ42 (median 5.3, *p* = 0.03) and Aβ40 (median 6.2, *p* = 0.045, Fig. 4a). Similarly, the percentage of Aβ43-affected leptomeningeal vessels (median 5.6) was significantly lower compared to Aβ42 (median 17.3, *p* = 0.007) and Aβ40 (median 27.4, *p* = 0.003). The percentage of leptomeningeal vessels in which Aβ42 was detected was significantly lower than the percentage of Aβ40-affected vessels (*p* = 0.012) (Fig. 4b-e).

### Peptide length correlates to Aβ load

**Fig. 5:**
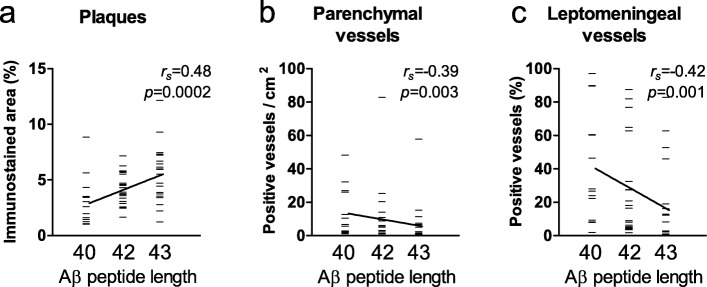
Plaque and CAA severity correlate to Aβ peptide length. Analysis of plaque and CAA severity in brain tissue of AD patients immunostained for Aβ43, Aβ42, and Aβ40 shows that Aβ peptide length correlates to plaque load (**a**). An inverse correlation between Aβ peptide length and both the number of Aβ-affected parenchymal vessels (**b**) and leptomeningeal vessels (**c**) is observed

A correlation was observed between Aβ peptide length and Aβ load in plaques (Aβ43 > Aβ42 > Aβ40, *r*_*s*_ = 0.48, *p* = 0.0002, Fig. 5a). Interestingly, an inverse correlation was observed between Aβ peptide length and the numbers of both parenchymal (Aβ40 > Aβ42 > Aβ43, *r*_*s*_ = − 0.39, *p* = 0.003, Fig. 5b) and leptomeningeal (*r*_*s*_ = − 0.42, *p* = 0.001, Fig. 5c) vessels positive for the different Aβ isoforms.

### Effect of immunotherapy on Aβ peptides in plaques

**Fig. 6:**
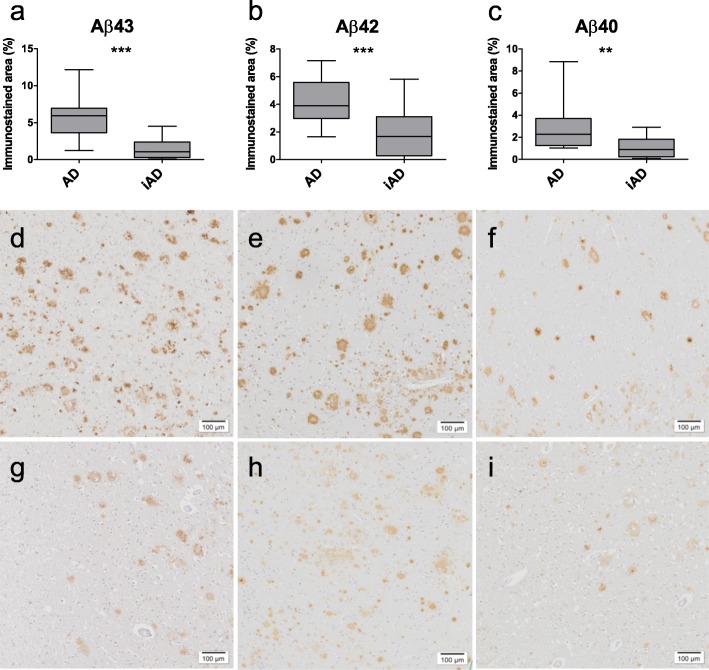
Effect of immunotherapy on parenchymal Aβ load. Aβ43 (**a**), Aβ42 (**b**), and Aβ40 (**c**) loads are significantly lower in iAD cases compared to non-immunized AD cases. Representative images of Aβ43 (**d, g**), Aβ42 (**e, h**), and Aβ40 (**f, i**) load in AD (**d-f**) and immunized AD cases (**g-i**). Box plots show median values with the 25th and 75th percentile as boundaries and whiskers indicating minimum and maximum values. Scale bar = 100 μm. **p ≤ 0.01; ***p ≤ 0.001

Quantification of Aβ in the iAD cases showed a reduction in median immunostained area values by a factor of 5.5 for Aβ43 (AD = 6.0; iAD = 1.1; *p* < 0.0001), a factor of 2.3 for Aβ42 (AD = 3.9; iAD = 1.7; *p* = 0.0006), and factor of 2.5 for Aβ40 (AD = 2.3; iAD = 0.9; *p* = 0.004), compared to the unimmunized AD cases (Fig. 6a-i).

### Effect of immunotherapy on Aβ peptides in CAA

**Fig. 7:**
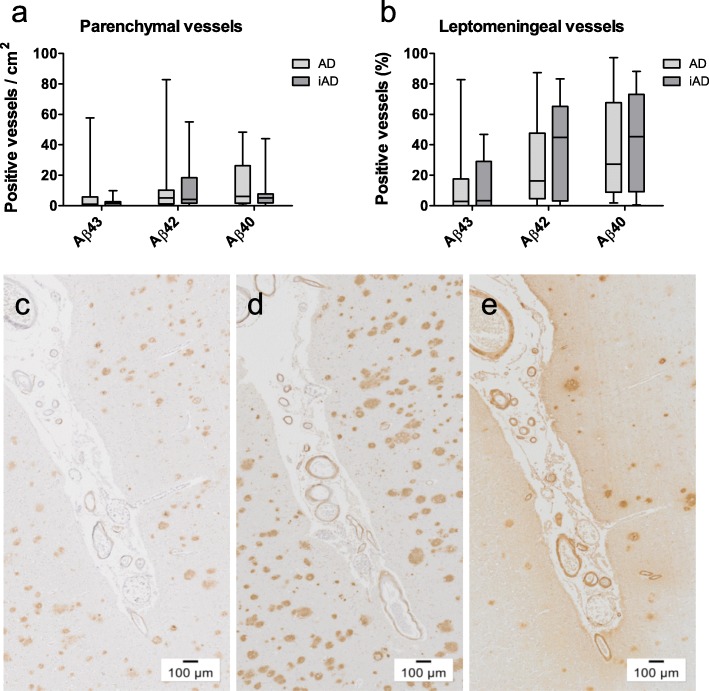
Effect of immunotherapy on cerebrovascular Aβ load. The numbers of parenchymal (**a)** and leptomeningeal (**b)** vessels affected by Aβ43, Aβ42, or Aβ40 did not differ between AD and iAD cases. Images of cerebrovascular immunostaining of Aβ43 (**c**), Aβ42 (**d**), and Aβ40 (**e**) in the same cortical area of an immunized AD case. Box plots show median values with the 25th and 75th percentile as boundaries and whiskers indicating minimum and maximum values. Scale bar = 100 μm

**Fig. 8:**
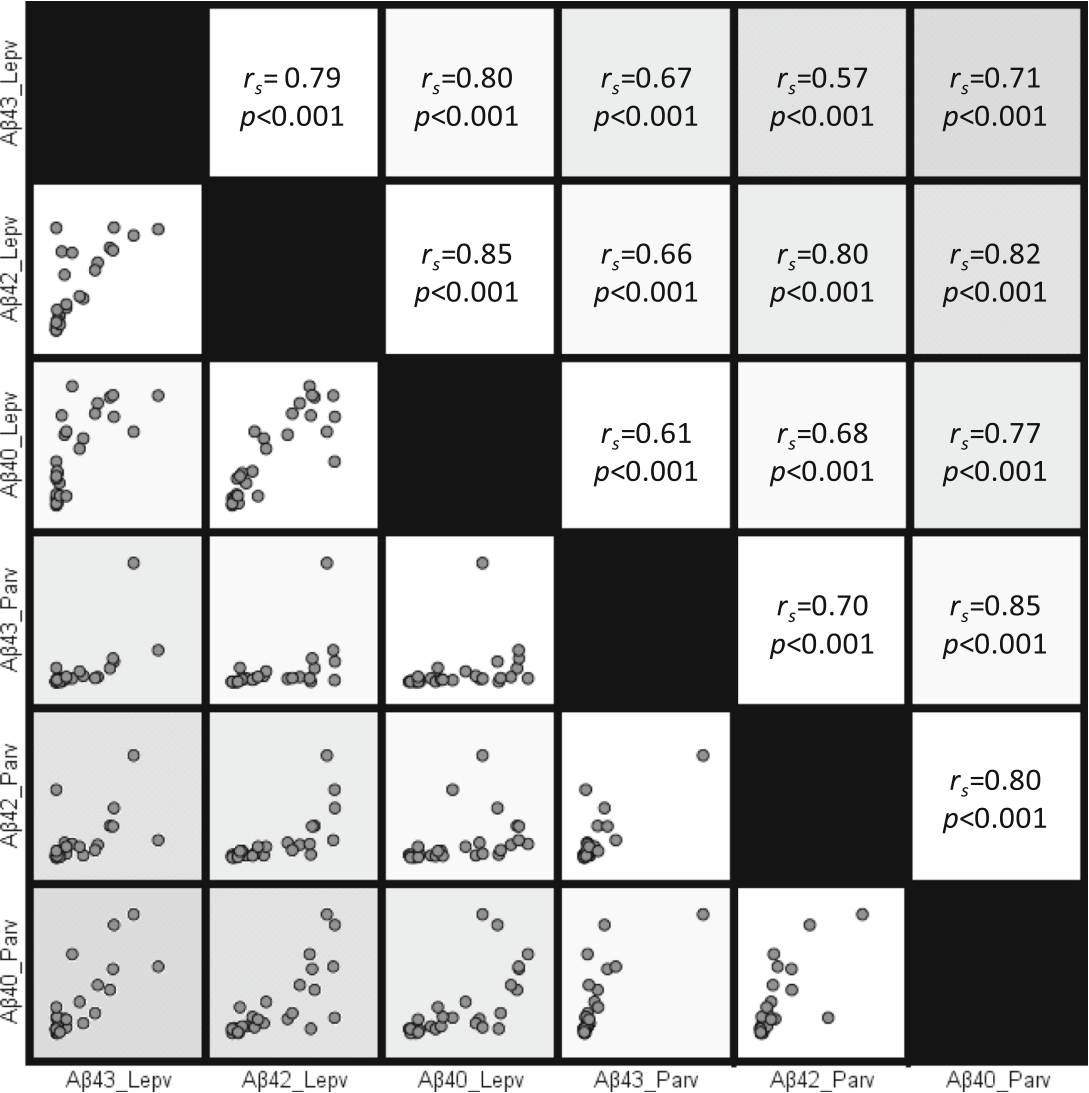
The numbers of vessels affected by the different Aβ isoforms strongly correlate to each other. Parv = parenchymal vessels; Lepv = leptomeningeal vessels

There was substantial variation in the numbers of Aβ-positive vessels between iAD cases. No significant differences for the numbers of parenchymal vessels affected by Aβ43, Aβ42, and Aβ40 were detected between non-immunized and immunized AD cases (Fig. 7a). Similarly, no differences were observed for the percentages of stained leptomeningeal vessels. Median values of positive leptomeningeal vessels were 45.4% in iAD versus 27.4% in AD for Aβ40, and 45.0% in iAD versus 16.4% in AD for Aβ42, whereas they were almost identical for Aβ43 (3.4% versus 2.9%). Analysis of the complete cohort (combining AD and iAD cases) showed that the numbers of leptomeningeal or parenchymal vessels affected by either Aβ43, Aβ42, or Aβ40 were strongly correlated to each other, with *r*_*s*_ values between 0.57 and 0.85, and *p* < 0.001 for all correlations (Fig. 8). These correlations were also observed in separate correlation analysis of the unimmunized AD cohort (*r*_*s*_ values between 0.63 and 0.88, and *p* values between < 0.001 and 0.015). Similarly, 14 out of 15 peptide correlations were significant in the immunized AD cohort (*r*_*s*_ values between 0.55 and 0.85, and *p* values between < 0.001and 0.04), the relation between leptomeningeal Aβ43 and parenchymal Aβ42 being the only exception (*r*_*s*_ = 0.44, *p* = 0.11).

## Discussion

In this study, we have explored the relatively little studied Aβ43 peptide and compared its cerebral expression levels in AD cases to those of Aβ42 and Aβ40, and studied the effects of active Aβ42 immunotherapy. Aggregation propensity [15] and neurotoxic properties [8] of Aβ43 have been defined as indicators for an important pathogenic role of Aβ43 in AD. Knock-in mice containing a presenilin-1 R278I mutation overproduced Aβ43, and crossing these mice with APP transgenic mice resulted in offspring with elevated Aβ43 levels, impaired short-term memory, and accelerated amyloid pathology [29]. Our results in AD cases also indicated that large amounts of Aβ43 accumulated into plaques, to a degree that is similar to that of Aβ42 accumulation. The cerebrovascular expression of Aβ43 has received sparse attention in literature. It has been demonstrated in brain tissue of five AD patients that Aβ43 and Aβ42 are absent from CAA [13]. Using mass spectrometry, it was demonstrated that Aβ43 levels in SDS preparations of brain tissue, containing blood vessels or parenchymal deposits other than plaque cores, are substantially lower compared to Aβ40 and Aβ42 [36]. Together, these data suggested that Aβ43 does not readily accumulate in the vasculature. In our study, however, we observed cerebrovascular accumulation of Aβ43 in AD, although less vessels were positive for Aβ43 compared to Aβ42 and Aβ40. Of note, despite low levels of cerebrovascular Aβ43, we observed a high abundance of Aβ43 in plaques. Interestingly, we found a correlation between Aβ peptide length and plaque load (Aβ43 > Aβ42 > Aβ40), indicating that longer Aβ peptides have an increased tendency towards accumulation in the brain parenchyma. In contrast, an inverse correlation was observed between Aβ peptide length and CAA load (Aβ40 > Aβ42 > Aβ43), suggesting that shorter peptides have an increased tendency to accumulate in the cerebrovasculature.

We found a higher plaque load of Aβ43 and Aβ42 versus Aβ40 (Fig. 2g), which likely drives the positive association between plaque load and peptide length (Fig. 5a). It is known that Aβ42 and Aβ40 differ in that Aβ40 is mainly located in plaque cores whereas Aβ42 is present also in the diffuse component of plaques [14], and the immunohistochemical pattern of Aβ43 in our study largely resembles that of Aβ42 (Fig. 2). It is possible, though, that a non-significant number of plaques contain more Aβ43 than the other isoforms, or that the distribution of Aβ43 extends beyond the area stained for Aβ42 in some plaques. This point has not been addressed in this study and requires meticulous analysis of expression patterns in individual plaques, which was outside the scope of this study. Similarly, it is likely that Aβ40, Aβ42, and Aβ43 are – a least to some extent – colocalised in blood vessel walls. Future studies including double or triple Aβ staining in brain tissue of AD and iAD patients are needed to provide insight into the exact co-localisation of various Aβ isoforms in plaques and CAA.

One hypothesis to explain the relatively low abundance of Aβ43 in the cerebrovasculature compared to other isoforms in AD, is a more efficient Aβ43 clearance at the BBB. Analysing the fate of Aβ43 following immunotherapy may provide insight in this potential efficient clearance of Aβ43. Similar to Aβ40 and Aβ42, the Aβ43 load in the parenchyma is decreased in response to Aβ42 immunotherapy. This is not surprising, as the majority of antibodies produced following AN1792 immunization are directed against the N-terminus of Aβ, which is shared by all three Aβ isoforms [19]. Interestingly, despite the absence of a gross significant difference in cerebrovascular expression of the three peptides in the two cohorts, Aβ43 was the only isoform that did not display the gross - yet not significant - increase of leptomeningeal CAA that was seen for Aβ42 (275%) and Aβ40 (165%) after immunization. This is noteworthy, considering the fact that Aβ43 is the isoform that was most rigorously removed from plaques (5.5 times lower Aβ43 load compared to a 2.3- and 2.5- fold reduction for Aβ42 and Aβ40 respectively), indicating that following immunotherapy Aβ43 is readily released from plaques. However, despite its increased availability for transport towards the vasculature, Aβ43 is less prone to accumulate at the vasculature than Aβ42. This suggests that Aβ43 might be more efficiently cleared at the BBB. A potential alternative explanation for the relatively low abundance of Aβ43 in the AD vasculature could be its high propensity to aggregate [15, 29], which may prevent Aβ43 from reaching the vasculature. There are, however, several arguments against that scenario. First, if its high aggregation propensity would explain the low abundance of Aβ43 in CAA, it would be expected that after solubilization of Aβ43 from plaques, its levels in CAA would increase. This is, however, not the case. Second, a high aggregation propensity does not necessarily prevent vascular accumulation of Aβ, as can be deduced from observations on the Dutch mutant Aβ40(E22Q) which is both very prone to aggregate and widely observed in the cerebrovasculature in mutation carriers [11, 18]. Therefore, the low abundance of Aβ43 in CAA seems to be more consistent with our hypothesis of a more efficient clearance of this Aβ isoform across the BBB.

We used immunohistochemistry to assess the antigen load of the different Aβ isoforms. Although this is a widely accepted method for relative protein quantification [6, 29, 30], a possible limitation of our study is that comparison of antigen load stained by different antibodies is not fully possible. However, by carefully selecting thresholds for every antibody, we ensured that only specific immunoreactivity for each antibody was captured, allowing for relative assessment of the expression of the different Aβ isoforms. However, immunohistochemistry does not yield a measure of absolute protein levels, and the observed differences between Aβ isoforms are relative.

Using immunoassays, we showed that the 21F12 antibody, often used for its assumed Aβ42-specificity, also recognized Aβ43, albeit more weakly. This observation has been shared and acknowledged by many researchers through the use of the term “Aβ42(43)-specific” when describing the 21F12 antibody. Many antibodies thought to be specific for Aβ42 also recognize longer variants, a fact that should be kept in mind when interpreting results obtained using such antibodies. We do not expect that this cross-reactivity has substantially affected our results, as the vascular and parenchymal changes of Aβ42 accumulation due to immunotherapy have been described before and our findings are in line with these previous studies [5, 23].

Earlier studies in our cohort have shown a decrease in the number of plaques and an increase of vascular Aβ42 and Aβ40 in the early years after immunotherapy [5, 30]. We confirmed significantly lower levels of Aβ42 and Aβ40 in plaques of iAD patients compared to unimmunized patients. However, we did not detect significantly higher numbers of Aβ42- and Aβ40-affected vessels in the immunized group. A possible explanation might be that in the current study only one brain region, the middle temporal gyrus, was included in the analysis, whereas in earlier publications multiple brain regions were studied, including the frontal, middle and temporal gyrus, and thus we might be less powered to detect these changes. Another, more important, explanation may be that, in the current study, we have expanded the iAD population with patients who survived longer after immunization than in previous studies. It is possible that the effects of active immunization on cerebrovascular Aβ levels may be transient or less pronounced in the long term. Indeed, we found indications of a negative correlation between Aβ42 levels in CAA and survival time (parenchymal Aβ42: r *=* − 0.605, *p* = 0.022; leptomeningeal Aβ42: r *=* − 0.629, *p* = 0.021), although the small number of cases precludes any firm conclusion.

Another potential confounder may be that, in line with previous observations, the extent of Aβ release from plaques is positively associated with the level of the immune response [12]. In addition, we observed a trend towards lower levels of Aβ in CAA in cases with a strong immune response (data not shown), indicating that, in addition to plaque removal, immunotherapy may lead to the removal of vascular Aβ, as has been demonstrated before in mouse models [3, 25]. However, again, the small number of cases and the limitation of only one studied brain region precludes us from drawing firm conclusions on these potential relations. Further studies on the long-term effects of active immunization on CAA levels are needed.

## Conclusions

We show that Aβ43 is not only an important constituent of plaques but also associated with CAA, albeit at lower levels than Aβ42 and Aβ40, suggesting that the expression pattern of Aβ pathology is isoform-specific. Furthermore, neuropathological analysis from the first human immunotherapy study also yielded preliminary evidence that Aβ43 may be more efficiently cleared at the BBB than Aβ42 and Aβ40, although mechanistic studies are needed to confirm this suggestion.

## Data Availability

The datasets used and/or analysed during the current study available from the corresponding author on reasonable request.

## Abbreviations

AD: Alzheimer’s disease
APP: Amyloid precursor protein
Aβ: Amyloid-β
BBB: Blood-brain barrier
CAA: Cerebral amyloid angiopathy
DAB: 3,3′ diaminobenzidine
OD: Optical density
ROI: Region of interest
RT: Room temperature
SWDBB: South West Dementia Brain Bank
TMB: 3,3′,5,5′-tetramethylbenzidine
